# Optimizing cardiorespiratory fitness after bariatric surgery – highly effective with very low adherence: HIT BAR randomized controlled trial

**DOI:** 10.1186/s13102-025-01307-y

**Published:** 2025-09-16

**Authors:** Alida Finze, Megan Duddek, Svetlana Hetjens, Erfan Ghanad, Christoph Reissfelder, Mirko Otto, Johanna Betzler, Christine Joisten, Susanne Blank

**Affiliations:** 1 Department of Surgery, Medical Faculty Mannheim, Universitätsmedizin Mannheim, Heidelberg University, Theodor-Kutzer-Ufer 1-3, 60167 Mannheim, Germany; 2 Department of Medical Statistics, Medical Faculty Mannheim, University of Heidelberg, Theodor-Kutzer-Ufer 1-3, 68167 Mannheim, Germany; 3 DKFZ-Hector Cancer Institute, University Medical Center Mannheim, Heidelberg University, 68167 Mannheim, Germany; 4 Institute of Movement- und Neurosciences, German Sport University Cologne, 50933 Cologne, Germany

**Keywords:** Bariatric surgery, Cardiorespiratory fitness, High intensity interval training, Roux-en-Y gastric bypass, Sleeve gastrectomy, Cardiovascular disease

## Abstract

**Background:**

Cardiovascular disease and obesity-related comorbidities are key factors addressed by metabolic-bariatric surgery (MBS). Although High intensity interval training (HIIT) has been proven effective in healthy cohorts, limited evidence exists regarding HIIT and adherence towards HIIT after MBS. This study aims to test feasibility and cardiorespiratory effect of HIIT after MBS.

**Methods:**

201 patients undergoing MBS were included in a four-week training protocol with 3 training groups (B-D) including different HIIT protocols on a bicycle designed for patients with obesity and one control group (A) at a university medical center in Germany. Ergometry with estimated VO2max, maximum blood lactate, maximum resistance, time spent on ergometer, and heart rate were performed prior to and after 4 weeks of training.

**Results:**

A significant effect of the four-week training could be shown through reduction of heart rate at 100 W, increase of maximum blood lactate, and maximum resistance when comparing the training groups to the control group (Δ 9,67 BPM; Δ 1.02 mmol/l; Δ 12 W respectively, all *p* < 0.05) However, adherence of the recruited patient group was very low, shown by a notably high drop-out rate of 78.1% overall (44 patients completed training). The majority of patients dropped out prior to the first training session.

**Conclusions:**

HIIT bicycle training in post-MBS patients is possibly very effective, however, adherence is extremely low. Although this study shows promising results, an effect on large patient groups cannot be expected if improvement of adherence and a wide range of training methods are not addressed first.

**Study registration:**

German Registry for Clinical Trials (DRKS) trial registration number DRKS00024939 on 20/09/2021.

## Introduction

Patients with obesity and metabolic syndrome have an increased risk of cardiovascular events and cardiovascular disease [1, 2]. Metabolic/Bariatric surgery (MBS) is currently the most effective available therapy for obesity and obesity-related diseases. Sleeve Gastrectomy (SG) and Roux-en-Y Gastric Bypass (RYGB) are the highest used MBS procedures with 62.5% and 28.5% of all primary MBS [3]. Through MBS we are able to achieve a significant weight loss as well as a significant reduction of cardiovascular events and cardiovascular risk factors [2, 4].

However, MBS cannot stand alone as a one-time therapy in this patient group. Apart from psychological interventions, reflection of eating behavior, and life-long supplementation of vitamins and calcium, regular physical activity after MBS is part of a multimodal treatment concept for MBS patients.

Previous studies have shown that physical activity interventions and exercise are effective after MBS and have the ability to increase weight loss, increase muscle strength, prevent bone loss, and increase VO2max [5–9]. However, the overestimation of adherence in this patient group has been seen as a major discussion point [10].

For a better understanding as to why this subject is very important, we emphasize that MBS should not be seen as a medical procedure performed by a surgeon on a patient alone. Treatment success relies substantially on surgery plus lifestyle changes the patient makes. These include nutrition, physical activity, and possible psychotherapy. However, specification of lifestyle change for physical activity is lacking in the current guidelines (including the IFSO consensus) [11]. Currently, an amount of 150–300 min of moderate to intense non-specified physical training per week is recommended, depending on the guideline [11, 12], in alignment with the WHO recommendations [13].

Regarding different types of training, high-intensity interval training (HIIT) has proven to be beneficial in terms of reducing cardiovascular disease and risk-factors in patients with obesity [14–16] and improving cardiorespiratory fitness and muscle mass preservation as well as quality improvement over a time span of 6–12 weeks or more [7, 17, 18]. Further, it has been proven to be safe and effective even in patients with chronic lung and heart disease, cancer and diabetes [19, 20]. After MBS, cardiorespiratory fitness plays an important role [21, 22], specifically in the reduction of cardiovascular events and risk factors as well as in improvement of health-related quality of life [23, 24].

To our knowledge, there has been no published HIIT protocol for patients directly after MBS as a structured training program for the entire patient cohort. For some subgroups (patients with sleeve gastrectomy and weight regain), HIIT has proven to be effective for weight loss and metabolic disease [25]. HIIT has been proven to be more effective in a short period of time than other training methods [26–28] and might therefore lower the threshold for attending training sessions regularly.

Choosing HIIT seems sensible due to the high proportion of patients with cardiovascular disease and diabetes in this specific training group. HIIT protocols are easily standardized and can therefore be compared well regarding outcome and feasibility. Further, they can be adjusted to patients’ needs [29, 30].

Cardiorespiratory fitness can be measured via ergometry and maximum blood lactate [31], VO2max [32, 33], and heart rate development at a steady workload [34, 35]. An increase in VO_2_max, a decrease in heart rate at a steady workload, and an increase in lactate tolerance correlate with an increase in cardiorespiratory fitness [32–35].

This study aims to test feasibility and adherence within a controlled setting of a HIIT protocol in patients undergoing MBS. As secondary endpoints we want to test the effect of HIIT with three different training programs on cardiovascular health such as heart rate upon physical stress, maximum blood lactate, and cardiorespiratory fitness. These secondary endpoints aimed to test the effectiveness of different HIIT protocols to build a base for a consecutive confirmatory trial.

## Materials and methods

### Study population

In this monocentric, randomized clinical trial, patients undergoing primary MBS (SG, RYGB and one-anastomosis gastric bypass (OAGB)) were screened between March 2021 and November 2023. Inclusion criteria were planned primary MBS, male and female patients between the age of 18 and 80 years, written consent, expected compliance, ability of riding a bike and physical fitness for riding a bike, expected available time frame and travel opportunity for bicycle training at the screening center and body mass index (BMI) ≥ 35 kg/m^2^ with associated disease or BMI ≥ 40 kg/m^2^ according to the current German guidelines for metabolic and bariatric surgery. Exclusion criteria were language barrier, expected non-compliance, pregnancy or breastfeeding, physical impairment not allowing bicycle training or physical activity, exacerbated lung- or heart disease and missing ability to consent. Patients were contacted via phone and email for the training sessions. Due to the pilot character of this trial, methods were not changed during the trial. Patient cohort size was determined by a statistician as a calculation of likely similarity between the intervention groups ajar to the local distribution of surgical procedures, gender, and BMI.

This trial was terminated early due to the extremely high drop-out rate and therefore prolonged recruitment period (see results section) in agreement with the principal investigator of the study.

The trial was approved by the local ethics committee prior to launch. The trial was registered in the German registry for clinical trials (DRKS) with the registration number DRKS0002493 as the HIT BAR trial.

### Baseline data

Visit 1 consisted of inclusion into the trial at a routine outpatient clinic visit of the patients when giving written consent to surgery before surgery. Patients were consulted about current medication, pain, eating habits (liquid diet, soft diet, solid foods), hours of physical training per week, and current symptoms (nausea, vomiting, bowel habits, and fatigue) and baseline data was obtained. The questionnaire was designed specifically for this study and has not been used elsewhere.

The same data was obtained in visits 2 and 3 prior to ergometry. In addition, resting blood pressure and heartrate were determined.

### Determining cardiorespiratory fitness

Before (Visit 2, also referred to as pre-training) and after the training period (Visit 3, also referred to as post-training), a bicycle ergometry test was performed. Patients had a warm-up period of 5 min at 50 W (W) and then started the ergometry at 50 W. Every minute, the resistance was increased by 10 W. This specific protocol (smaller resistance increase steps than used for athletes) was chosen for better distinction of maximum power in a patient group with expected low adherence. Patients were advised to ride the bike to exhaustion. Ergometry was also ended when adverse events occurred, or the heart rate reached a level > 220 beats per minute (bpm) minus patient age. During the ergometry, heart rate was determined via a Polar^®^ H9 M-XXL Chest Band (Kempele^®^, Finland). Blood lactate was determined at rest and every 3 min during the ergometry as well as at exhaustion. Lactate was measured via prick on the earlobe with a Lactate Scout 4 device (EKF^®^, Germany). Maximum heart rate, heart rate at every intensity level, maximum resistance (Watt) and time spent on ergometry were measured. VO2max was then approximated for each patient using the following formula:

VO_2_max(L x min^-1^) = ([(HRindex x 6)-5.0] x (3.5 body weight (kg))) [36]

**Fig. 1:**
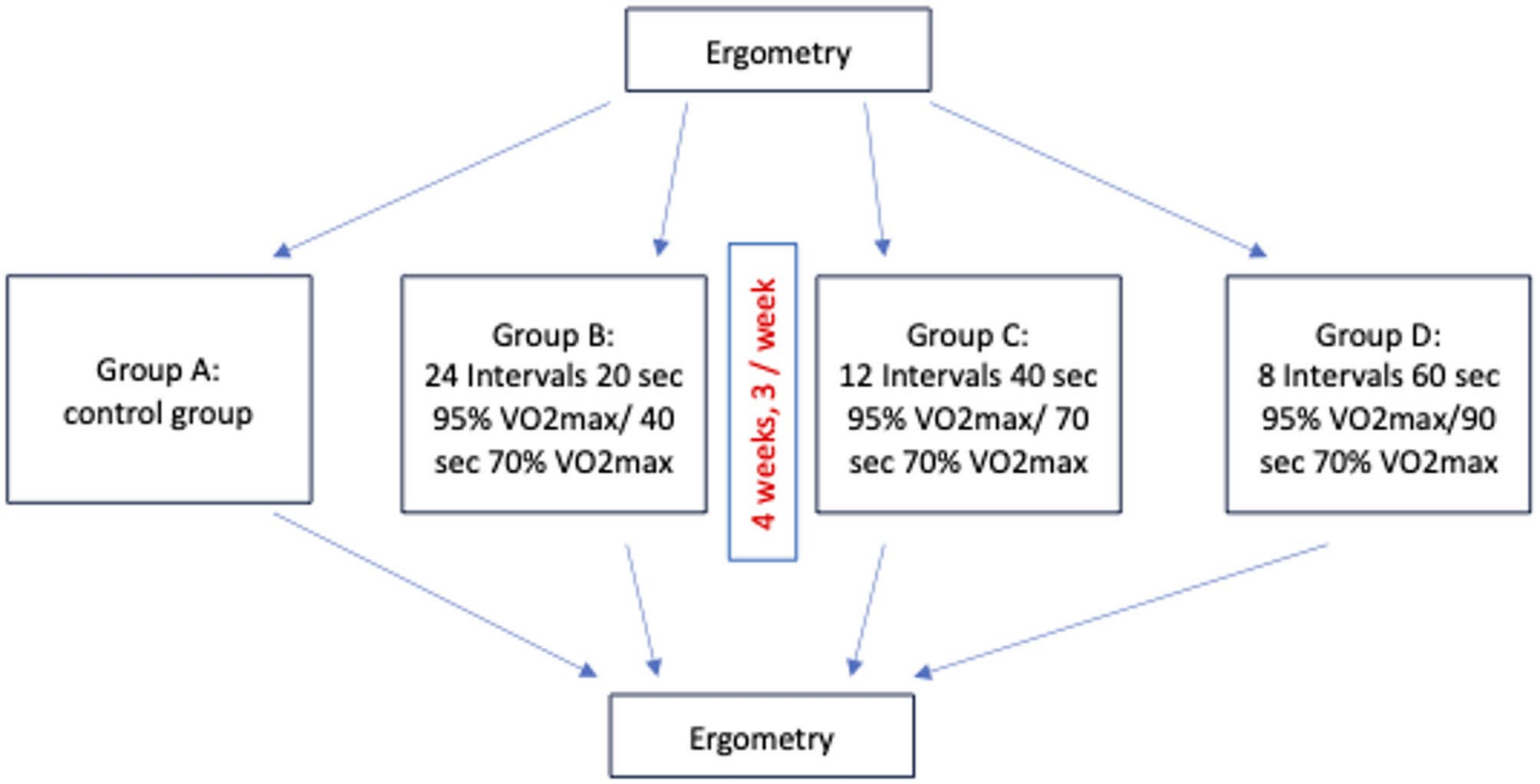
sec = seconds; VO2max = estimated maximum oxygen intake per kg bodyweight. Training was performed minimum 2 times per week and maximum 3 times per week

The approximated VO_2_max was used for the training protocols (Fig. 1). If patients were not able to complete the protocols repeatedly, intensity was decreased. If patients rated intensity during training as low and consented, intensity was increased between the training sessions.

### Training/Protocol

Patients were randomized through a randomization list assigning intervention groups without restrictions created by a statistician in advance into one control group (A) and three training groups (groups B, C, and D) after surgery. Sequentially numbered containers were used for patient inclusion. Patients were enrolled and assigned to an intervention group by a physician assigned to the trial and then followed up by an assigned doctorate student and physicians assigned to the trial. Patients were advised to continue any additional sport program during the trial that they wanted to pursue within training and control groups. No further guidance was given for daily activities or other sports to maintain realistic conditions. The training period consisted of a four-week training program within 8–12 weeks after surgery using a high intensity interval training on a bicycle-ergometer especially designed for patients with obesity (928 E, Monark^®^ Exercise AB, Sweden). This specific time frame was chosen to guarantee complete recreation from surgery without increased risk for trocar herniation and to increase adherence in case of sickness, childcare issues, or vacation. The limit was set to 12 weeks after surgery due to this window being especially sensitive towards losing muscle mass after MBS [37]. Trainings were performed three times a week, a minimum of 8 trainings had to be completed for a successful completion of the trial. The training protocols are displayed in Fig. 1. Training protocols were designed according to preexisting HIIT protocols according interval duration and intensity [29, 30].

If patients could not attend at least 8 training sessions in total, the trial was rated as “not successful” for the patients. During each training, intensity of the training was rated by the patients via Borg Scale (rating from 0 to 10, representing no exhaustion to maximum exhaustion, respectively) [38]. The training sessions lasted around 30 min of time (Fig. 1), training sessions were adapted to the patients’ needs if necessary. If training session intensities were adapted but completed, they were rated as successful.

Every training session for each training group included a five-minute warm-up and a 5-minute cool-down at 50 W. The training protocols were designed as follows:

Group B: 24 intervals of 20 s with 95% VO2max in alternation with 40 s with 70% VO2max.

Group C: 12 intervals of 40 s with 95% VO2max in alternation with 70 s with 70% VO2max.

Group D: 8 intervals of 60 s with 95% VO2max in alternation with 90 s with 70% VO2max.

### Data analysis

Analysis was done in the per-protocol population as outcome data (visit 3) was not available for the intention to treat population. Statistical analysis was performed with the support of a statistician using SAS^®^ release 9.4. Quantitative data was analyzed using mean with standard deviation. For comparison of groups, Chi^2^ and Fischer tests were used. When comparing two groups. If data was normally distributed, T-tests were used. If data was not distributed normally, Mann-Whitney U-tests were used. For more than two groups, ANOVA was used. For adjustment of *p*-values, Scheffé Test was used. Results were significant if *p* < 0.05. A sample size calculation was not possible due to the pilot character of this study.

## Results

### Baseline data

Of the 44 patients included in the per-protocol analysis, the mean age was 42.39 years (SD 9.41) with a mean BMI of 46.75 kg/m^2^ (SD 7.22). Overall, 12 men with a mean BMI of 48.56 kg/m^2^ (SD 6.11) and 32 women with am mean BMI of 46.07 kg/m^2^ (SD 7.57) were included (*p* = 0.27 for difference in BMI). Of the included cohort, 27 patients received RYGB, 16 patients received SG, and one patient received OAGB. There were no significant differences between the groups in age, comorbidities or BMI. Details are displayed in Tables 1 and 2. All patients were on a solid diet.

**Table 1:** Baseline data groups A-D

Baseline Data	Group A (*n* = 14)	Group B (*n* = 9)	Group C (*n* = 11)	Group D (*n* = 10)	*p*-Value
male: female (%)	4:10 (28.6:71.4)	1:8 (11.1:89.9)	3:8 (27.3:72.7)	4:6 (40:60)	0.6344
Gastric Bypass	9	6	7	6	1
Sleeve Gastrectomy	5	3	4	4	
Mean Age (SD)	42.29 (7.92)	42 (8.17)	39.82 (9.70)	45.7 (12.16)	0.5713
Mean BMI (SD)	49.55 (7.1)	45.64 (7.98)	45,52 (7.81)	45.16 (5.82)	0.3838

SD = Standard Deviation; Gastric Bypass includes all Roux-en-Y Gastric Bypass apart from 1 One-Anastomosis Gastric Bypass

**Table 2:** Comorbidities of included patients

Comorbidity	*N* (%)
Cardiac disease	22 (50)
Lung disease	7 (15.91)
History of malignant tumor	1 (2.27)
Musculoskeletal	26 (59.09)
Diabetes mellitus Type II	6 (13.64)
Psychiatric	11 (25)
Dyslipidemia	13 (29.54)
Obstructive Sleep Apnea	24 (54.55)
Other	32 (72.7)

Comorbidities of the included patients. Cardiac disease includes arterial hypertension

Of the included patients that completed the trial, 4 patients had regular medication intake of beta-blockers and/or calcium-channel antagonists. These were distributed as follows: 1 patient from group B, 2 patients from group C, and one patient from group D. The medication intake was not statistically relevant regarding heart rate.

The groups were further distinguished between patients that completed the trial (completers) and patients that did not complete the trial (non-completers). Data was available for 139 patients that did not complete the trial and 44 patients that completed the trial. There were no significant differences between the two groups (including Cardiac disease, lung disease, Diabetes). Some relevant data for possible termination for completers vs. non-completers is displayed in Table 3.

**Table 3:** Baseline characteristics of completers vs. non-completers

Comorbidity	Completers mean (SD)	Non-Completers mean (SD)	*p*-Value
BMI (kg/m^2^)	47.26 (7.33)	46.75 (7.22)	0.6986
Age (years)	40.38 (11.48)	42.49 (9.41)	0.3130
Pain (NAS)	2.76 (3.15)	2.14 (2.9)	0.2834
	**N (%)**	**N (%)**	
Sex m: f	12:32	22:73	0.5996
Dyspnea	3 (6.82)	13 (13.68)	0.2381
Physical Weakness	4 (9.09)	19 20.0)	0.1074
Fatigue	25 (56.82)	45 (47.37)	0.3000

Baseline characteristics of patients that completed the trial (completers) vs. patients that did not complete the trial (non-completers) are displayed. Mean values are displayed, there were no significant differences. NAS = Numeric Analogous Scale for pain from 0 (no pain) to 10 (worst pain possible). The second part of the table refers to baseline data presented as percentages. M = male, f = female

### Early withdrawal and drop-out rates

Overall, 620 patients were screened, 201 patients were included in the study and gave written informed consent prior to surgery.

**Fig. 2:**
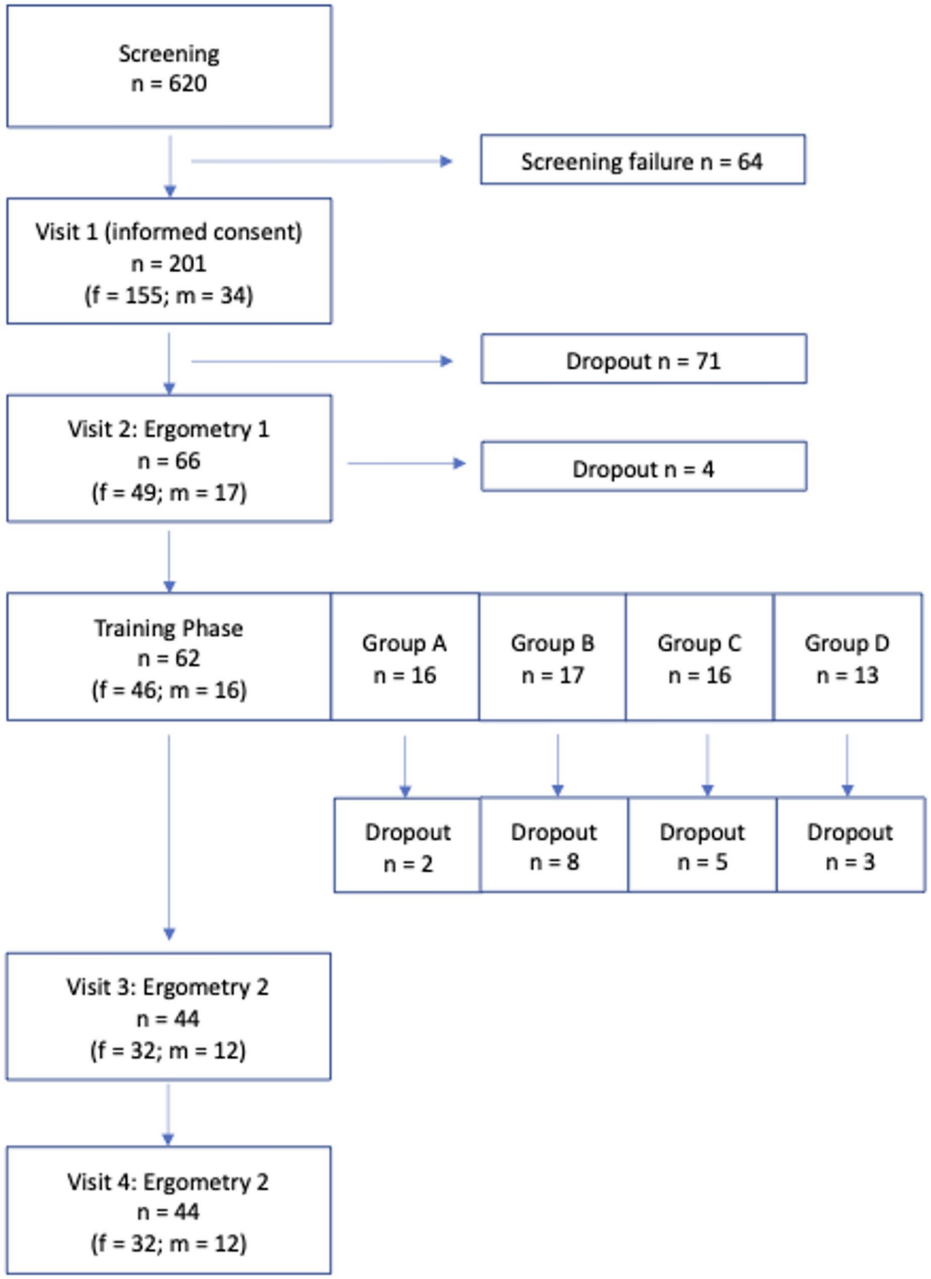
Flow chart of recruitment, screening failure and drop outs in each training group

In summary, 64 patients dropped out of the study after inclusion and prior to randomization. Overall, 137 patients were randomized. After randomization, 71 patients withdrew from the study early, 4 patients after visit 2 and 18 patients withdrew from the study during the training phase (29% of the patients who started the training). A total of 44 patients completed the trial and were included in the per-protocol-analysis. Overall, the drop-out- rate was 78.1%. Due to high drop-out rate, the study was ended early (initial goal for completion of visit 2: 88 patients). After randomization, drop-out rates were as follows in the different groups: Group A (Control group) had a drop-out rate of 46.1%, group B had a drop-out rate of 75.7%, group C had a drop-out rate of 70.3%, and group D had a drop-out rate of 73% after randomization (Fig. 2).

When comparing training protocols regarding drop-out, the largest number of patients withdrew from the study during training phase when training in group B. 47% of patients dropped out during training within group B, 31,3% of patients dropped out during training phase in group C, and 23.1% of patients withdrew from the study during training phase in group D.

Patients were not obliged to give information for withdrawal of the study. During the training phase, few patients dropped out due to sickness. 4 patients withdrew from the trial during the first ergometry due to knee pain or physical incompatibility with the bicycle. In the patient group terminating the trial prior to the first ergometry, the main reason for withdrawal was stated to be a lack of time for physical exercise during daily routine. Most patients were not willing to give information regarding reasons for withdrawal. (Fig. 2)

No major adverse events were reported. 8 mild adverse events such as leg and knee pain and short-term shortness of breath resolved within minutes and did not cause long-term damage.

### Performance in ergometry

There were no differences over time in estimated VO2max. However, delta of time spent on the ergometer until exhaustion was higher in patients of group B, C, and D than in patients of the control group when comparing ergometry pre-training and post-training. The training groups were able to perform ergometry longer until exhaustion than the control group. The control group presented with a Δ of 55.57 s. Group B increased the time spent on ergometer by Δ121.22 s, group C by Δ119.36 s and group D by Δ159.8 s between visits 2 and 3 (p all < 0.05).

**Fig. 3:**
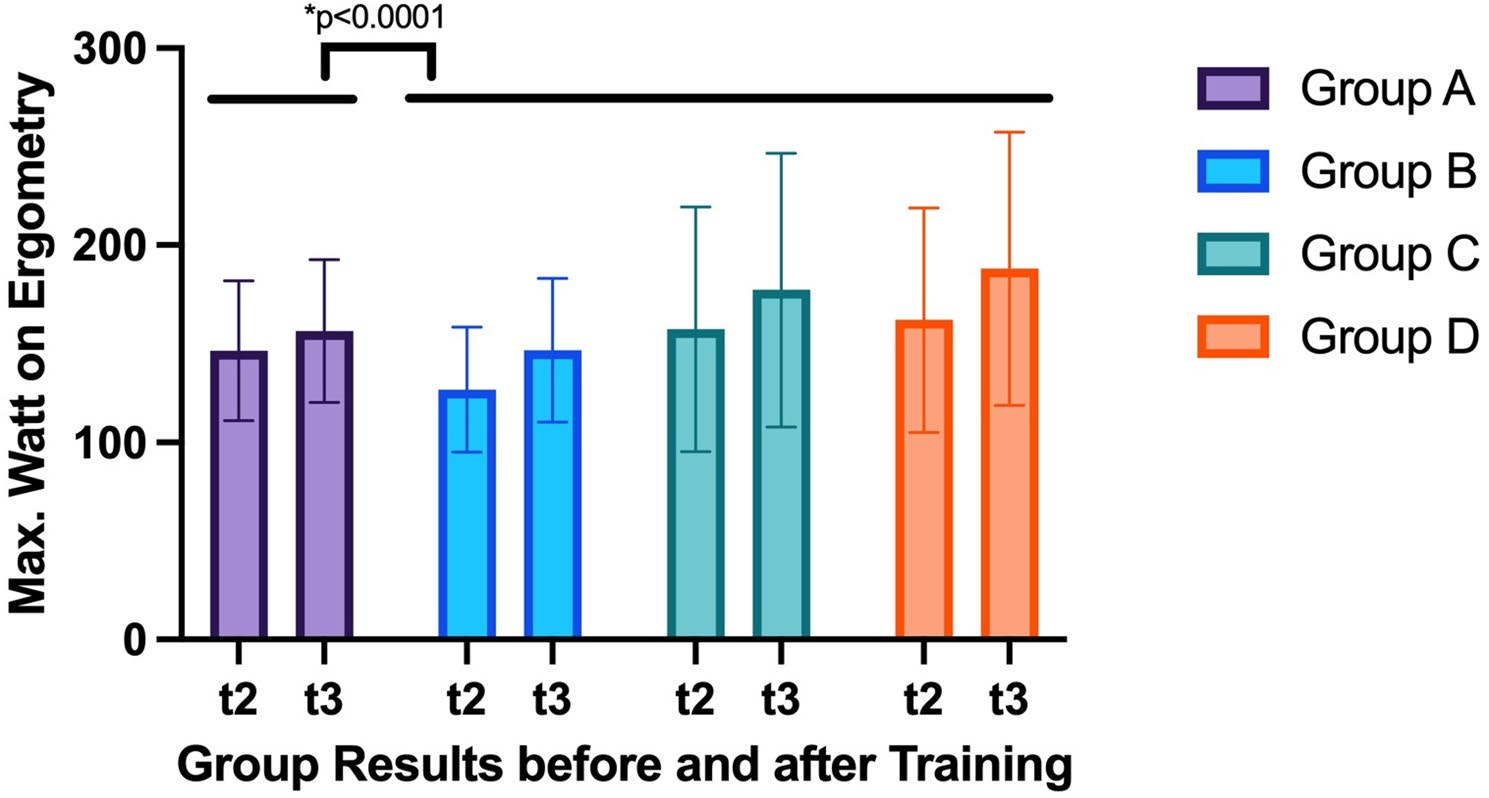
t2 = visit 2; t3 = visit 3; * = significant *p*-value. The training groups combined were able to achieve significantly higher Watt levels in ergometry at visit 3 than the control group

Groups B, C, and D were able to reach significantly higher resistance (in Watts) at the second ergometry than the control group (*p* < 0.0001). Each group individually reached significantly higher resistance levels during the second ergometry (Control group: *p* = 0.0072 with Δ 10 W; Group B: *p* = 0.0012 with Δ 20 W; Group C: *p* = 0.0006 with Δ 20 W; Group D: *p* = 0.0004 with Δ 26 W), however, the training groups showed significantly higher increase of Watt performance during ergometry than the non-training group (Fig. 3).

There were no changes or differences in Borg’s Scale over time and between the training groups.

### Changes in heart rate, weight, VO2max, and blood pressure

**Fig. 4:**
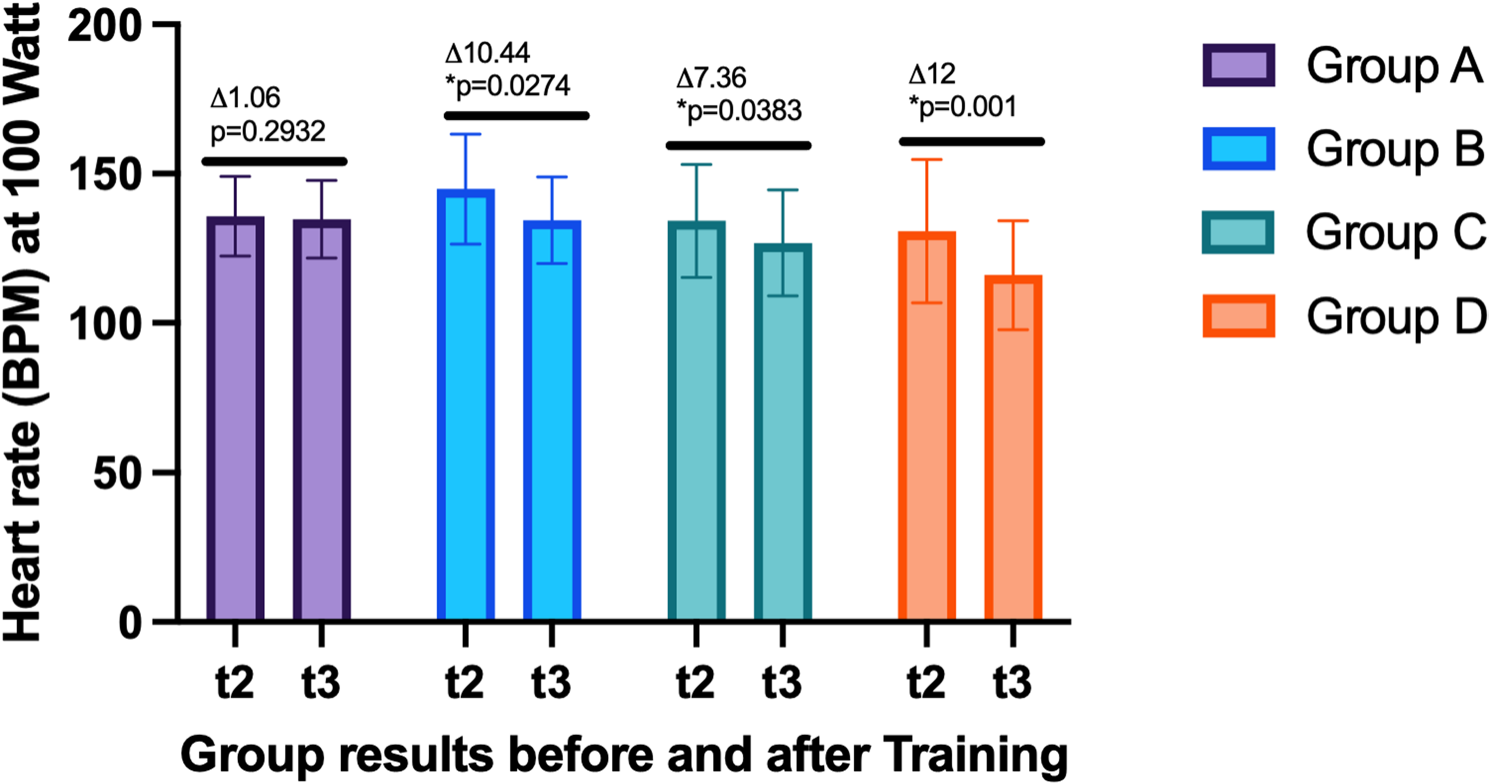
t2 = visit 2; t3 = visit 3; * = significant *p*-value. Significant reduction of heart rate at 100 W on the ergometer could be seen in groups B, C, and D, no significant reduction could be seen in the control group

Significant changes could also be seen in measured heart hate of the patients at fixed resistance points within the ergometry (analysis was performed at 90 W, 100 W, and 110 W resistance). Overall, the training groups had significantly better heart rate reduction over time than the control group. Results are shown exemplarily for 100 W in Fig. 4. For 100 W, a Cohen’s D was calculated and showed a small to medium effect of 0.354.

At 110 W, with *p* = 0.0396 it could be shown that group D had the best results in reduction of heart rate between visits 2 and 3. Group D had a mean reduced heart rate by 13.89 bpm, whilst groups A, B, and C were reduced by 2.65, 5.83, and 6.22 bpm, respectively.

There were no significant differences in total weight loss at visits 2 and 3 between the patient groups A, B, C, and D. Further, no significant change in systolic or diastolic blood pressure could be shown within the groups and when comparing the control group to groups B, C, and D at visits 3 and 4. Significant change in resting heart rate could also not be seen. There was no significant change in estimated VO2max over time and when comparing the control group to groups B, C, and D at visits 3 and 4.

No differences between patient groups were seen when comparing blood samples and symptoms.

### Maximum blood lactate

**Fig. 5:**
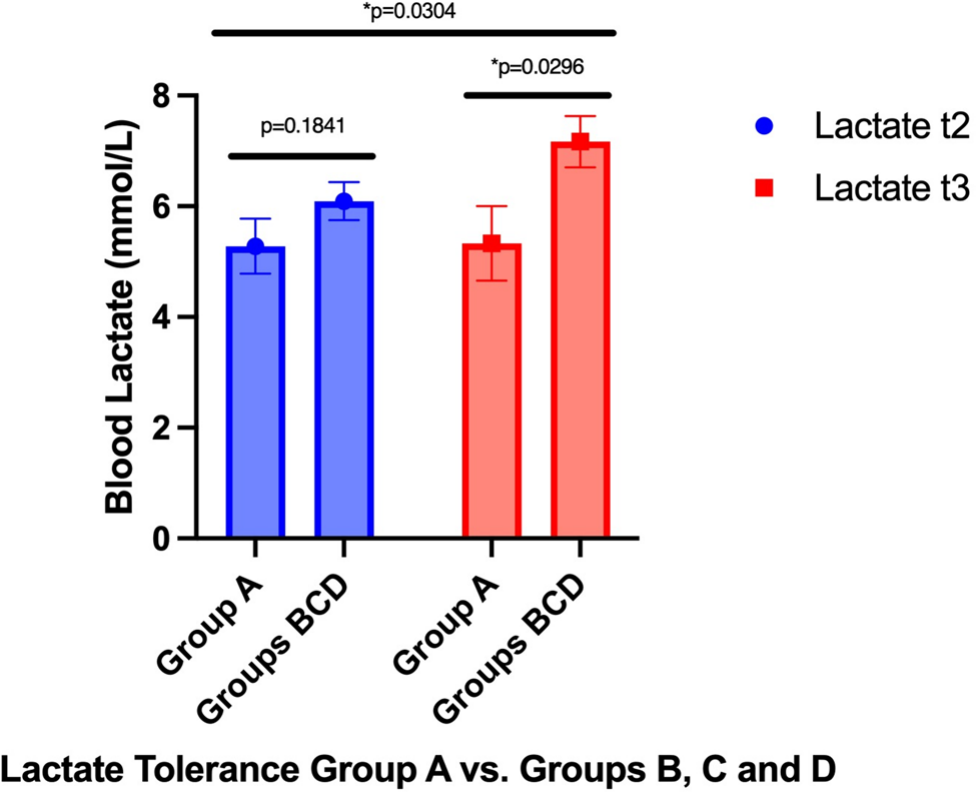
t2 = visit 2; t3 = visit 3; * = significant *p*-value. The bars indicate maximum blood lactate over time between visits 2 and 3 and comparing training (Groups BCD) to non-training (Control group)

Maximum blood lactate was measured after finishing ergometry in visits 2 and 3 in each patient. Overall, the control group had lower maximum blood lactate than groups B, C, and D. Maximum lactate in the control group changed from 5.28mmol/l (SD 2.01) pre-training to 5.33mmol/l (SD 2.10) in visit 3 (*p* = 0.8966). Groups B, C, and D changed from 6.42mmol/l (SD2.10), 5.69mmol/l (SD 1.75), and 6.24mmol/l (SD 1.66) to 7.09mmol/l (SD 2.98), 7.10mmol/l (SD 2.44), and 7.31mmol/l (SD 2.95), respectively (*p* = 0.4249; *p = 0.0332; *p* = 0.3159, respectively). Over time, maximum blood lactate increased in combined groups B, C, and D, while it did not increase in the control group (*p* = 0.0304). Details are shown in Fig. 5. Cohen’s D was calculated and showed a medium to large effect of -0.593 for the control group vs. all training groups.

## Discussion

Taking under consideration the high drop-out rate, adherence to HIIT in the short term after MBS is a relevant limitation and feasibility cannot be declared. Still, in alignment with preexisting data [25], this study demonstrates that HIIT training after bariatric surgery improves fitness over a time span of only four weeks of training. Previous data has shown that HIIT is possible prior to surgery and effective in short term for treatment of obesity [39] and also effective for improvement of cardiorespiratory fitness in case of weight regain after MBS [25]. Although exercise seems to be subject of current research [40], limited evidence exists describing feasible training protocols after MBS.

Cardiorespiratory fitness is essential for a long-term and effective treatment of obesity, cardiovascular disease and other obesity-related diseases. In addition, physical training as a preventive method against sarcopenia after MBS is expected to gain relevance regarding to the currently young MBS patient group when they reach an age of 60–80 years.

The rationale for our trial was the possible maximization of the effect of MBS on cardiorespiratory risk and disease. Both increase of lactate threshold, being closely correlated to greater cardiorespiratory fitness [41, 42], and a similar effect of heart rate and maximum power [35, 43] could be reached within the brief study period. The recommended minimum training time for visible and sustainable effects is 8–16 weeks [29, 30]. Nevertheless, a four-week period with a small patient group was sufficient for significant improvement in cardiorespiratory fitness and therefore underlines the high potential of supervised training programs after MBS. Taking into account that the control group was asked to continue any planned sports program and was informed about current recommendations of 150–300 min of physical activity per week [44], the high effect of the relatively short intervention time of bicycle HIIT for 60 to 90 min per week from this study seems very promising. However, this short period of time or the estimation method might have led to the lack of improvement in VO2max. For VO2max, no clear conclusion can be drawn.

A clear limitation of the study was low compliance and high drop-out. However, in our opinion, this is also one of the most important conclusions that can be drawn from this study for development of future clinical trials. The early withdrawal rate within the trial was almost 80%. The inclusion to the trial was done prior to surgery and at that time point patients confirmed the ability to participate to the trial regarding time, travel and physical impairment. Patients who had rated their own motivation for physical exercise as too low for participation or did not want to train on a bicycle prior to surgery were not included in the trial due to expected non-compliance and to minimize bias from preference of types of physical activity. The decrease of motivation after surgery might be associated to a deficit in patient education but could be due to a non-suitability of HIIT training in the short term after surgery. Additionally, the investment with respect to time and also travel costs was quite high for patients not living in the surrounding area and travel costs increased significantly during the time span of the trial. It should be considered that impaired physical function might be a factor leading to high withdrawal rates. However, patients with known physical impairment were excluded from the trial and only 4 patients dropped out during the trial due to physical impairment. These findings align with previous data stating that lack of lifestyle change and physical activity is most likely not fully explained by physical impairment [45, 46].

Missing out on physical activity after or before bariatric surgery might have additional multifactorial causes, including weight-stigma and concluding self-exclusion from sport programs [47]. However, this trial included a safe environment with one-on-one training supervision without competitional aspects in a room not accessible to others during the training phases and therefore minimizes room for stigma and comparison to other people.

Overall, patients were reminded about the importance of physical activity and possible consequences. Training was shortened down to a time minimum and individualized according to every patient’s need. Patients were reminded to attend each session via phone prior to each session and sessions were offered to patients at any time from 7am to 9pm. Although we tried to create a low-threshold and low-stigma setting in the first place, it did not meet the needs of patients for a sufficient participation in a free-of-charge training program.

In further analysis, drop-out rates were highest between surgery and visit 2. The participation rate was higher prior to surgery and remained relatively high once training had started. However, it can be postulated that the group of patients that participated in the training might be a small, highly motivated group, which might result in a relevant selection bias. We do not suspect bias due to nutrition or lack of knowledge about the importance of physical activity: all patients were on a solid diet and advised towards an increased level of physical activity in concordance with the German guidelines.

The results of this study show us that HIIT might be an effective training, but that first of all we need to find ways to increase motivation, options, and offers for monitored physical activity after MBS in the first place. A less intense start and slow increase of training might also be beneficial for increased adherence and motivation. Monitoring physical activity after surgery has the potential of preservation of cardiovascular health and possibly long-term-prevention of sarcopenia in patients undergoing MBS. Increase of adherence might be providable for example via smart-phone applications in the future [48]. A lack of feedback of professional supervision, however, most likely leads to reduced physical activity [49].

Further training programs and protocols need to be explored regarding feasibility, effectiveness, and adherence for adequate recommendations and clear guidelines.

No factors likely to improve adherence could be identified during this trial. A higher percentage of patients dropped out in the training groups than in the control group than in the control group. However, differences were not significant.

Regarding the type of HIIT protocol, there were no significant differences between the training groups. However, the drop-out rate during the training phase was lowest in training group D with 23.1%. Also, there was a trend of an increased performance in Watt resistance during ergometry after four weeks of training in group D. This might suggest that a 60 sec 95% VO2max in combination with a resting phase of 90 sec at 70% VO2max might be the protocol with the highest adherence and the best results when compared to the other training protocols used in this study and can be used in future studies. However, since the included patient number was very low, further research is needed before an adequate recommendation regarding training protocol can be made. This is reflected in the effect strength calculated in our study. Other types of training should also be considered for integration in future study protocols. For future trials, measurement of true VO2max should be considered. This trial used an estimated calculated VO2max as reference.

## Conclusion

Concluding, this analysis showed a high potential in improvement of cardiorespiratory fitness after MBS in the subgroup of motivated patients. Adherence is currently insufficient to incorporate HIIT into standard care. Supervision and improvement of adherence and compliance is crucial and needs to be addressed. Resulting from the low adherence prior to starting training as well as drop-out during the training period, focus should be laid on finding adequate training options with higher adherence after MBS.

## Data Availability

All data is available at University Medical Center Mannheim upon request. Data is not shared openly due to participant privacy as required by the local ethics committee.

## Declarations

### Previous presentations

Interim results were presented but not published at Deutscher Chirurgenkongress in April 2023.

## Abbreviations

BMI: Body Mass Index
BPM: Beats Per Minute
DRKS: Deutsches Register für klinische Studien (German Registry of Clinical Trials)
HIIT: High Intensity Interval Training
MBS: Metabolic Bariatric Surgery
OAGB: One Anastomosis Gastric Bypass
RYGB: Roux–en–Y Gastric Bypass
SD: Standard Deviation
SG: Sleeve Gastrectomy
VO2max: Maximum Oxygen Consumption
